# The stage-classified matrix models project a significant increase in biomass carbon stocks in China’s forests between 2005 and 2050

**DOI:** 10.1038/srep11203

**Published:** 2015-06-25

**Authors:** Huifeng Hu, Shaopeng Wang, Zhaodi Guo, Bing Xu, Jingyun Fang

**Affiliations:** 1 State Key Laboratory of Vegetation and Environmental Change, Institute of Botany, Chinese Academy of Sciences, Beijing, China; 2 Department of Ecology, College of Urban and Environmental Science, and Key Laboratory for Earth Surface Processes of the Ministry of Education, Peking University, Beijing, China; 3 National Satellite Meteorological Center, China Meteorological Administration, Beijing, China

## Abstract

China’s forests are characterized by young age, low carbon (C) density and a large plantation area, implying a high potential for increasing C sinks in the future. Using data of provincial forest area and biomass C density from China’s forest inventories between 1994 and 2008 and the planned forest coverage of the country by 2050, we developed a stage-classified matrix model to predict biomass C stocks of China’s forests from 2005 to 2050. The results showed that total forest biomass C stock would increase from 6.43 Pg C (1 Pg = 10^15^ g) in 2005 to 9.97 Pg C (95% confidence interval: 8.98 ~ 11.07 Pg C) in 2050, with an overall net C gain of 78.8 Tg C yr^−1^ (56.7 ~ 103.3 Tg C yr^−1^; 1 Tg = 10^12^ g). Our findings suggest that China’s forests will be a large and persistent biomass C sink through 2050.

Forest ecosystems contain over 80% of terrestrial vegetation carbon (C) and play a leading role in alleviating atmospheric CO_2_ elevation and stabilizing the global climate^1^,^2^. China ranks as the fifth largest country in terms of forest area and has the largest area of plantation in the world^3^. During the past decades, several studies have estimated biomass C storage and its changes in China’s forests, which detected a significant increase in the forest C stock since the mid-20^th^ century^4^,^5^,^6^,^7^,^8^,^9^,^10^. Furthermore, recent studies suggested that China’s forests have high potential to function as a C sink in the future because of the following characteristics^6^,^8^,^11^: (1) young age—more than one-third of the total forest area is covered by young-aged forests; (2) low average C density—the mean biomass C density of China’s forests is 41.3 Mg C ha^−1^ at present compared to 94.2 Mg C ha^−1^ of the world’s forests^1^; and (3) large area of planted forests—plantation area (40.0 × 10^6^ ha) accounts for 25.7% of the total forest area and is continually increasing. Despite these, little is known about the projected changes in C stock for China’s forests. Based on the relationships between forest biomass density and forest age for major forest types and China’s national forest inventory data between 1994 and 2003, Xu *et al*.^11^ first estimated the potential biomass C stocks of China’s forests during 2000–2050, suggesting a net increase of 7.23 Pg C during this period of 50 years. However, their approach does not take into account the effects of disturbance such as forest dieback and harvest, which potentially overestimates the capacity of China’s forests as a C sink. Therefore, it is important to develop more appropriate approaches to better evaluate the biomass C sequestration potential of China’s forests for the country’s emission reduction policy-making.

In population studies, a common approach to describe the dynamics of age- or stage-structured populations is to develop a matrix model, of which each element represents the probabilities that population in one age or stage class transfers to another (or itself) between two sequential periods^12^. Particularly, the stage-classified matrix models were developed to study the dynamics of stage-structured populations^13^, which have become effective tools for inferring life history parameters and assessing conservation strategies for specific species^14^,^15^. As forest inventory data are usually recorded by stage class, such matrix models also provide a promising framework to study regional- or continental-scale forest dynamics. Here, we use the stage-classified matrix approach to model the dynamics of China’s forests. We will first estimate the parameters of the stage-classified matrix with the recent updated forest inventory data, and then predict the changes in China’s forest biomass C stock between 2005 and 2050 using the developed matrix model.

## Results

### Parameter estimates for the transition matrix

Using the inventory data between 1994 and 2008, we estimated the stage-classified transition matrix for China’s forests (Table 1; also see Supplementary Table S1 online). For all stage classes, during two sequential periods, a major proportion stayed within the same stage class, and the probabilities of transition (to the older class) were small and decreased consistently with stage classes. More specifically, the transition probability for young-aged forests into mid-aged stage was 14.42%, and that for mature forests into overmature stage was only 1.44%. The largest dead or be harvested probability was found in mature forests (0.36%), followed by premature forests (0.13%), overmature forests (0.08%), mid-aged forests (0.03%), and young-aged forests (0.02%).

### Future area and biomass C stock of China’s forests in the next 45 years

**Figure 1:**
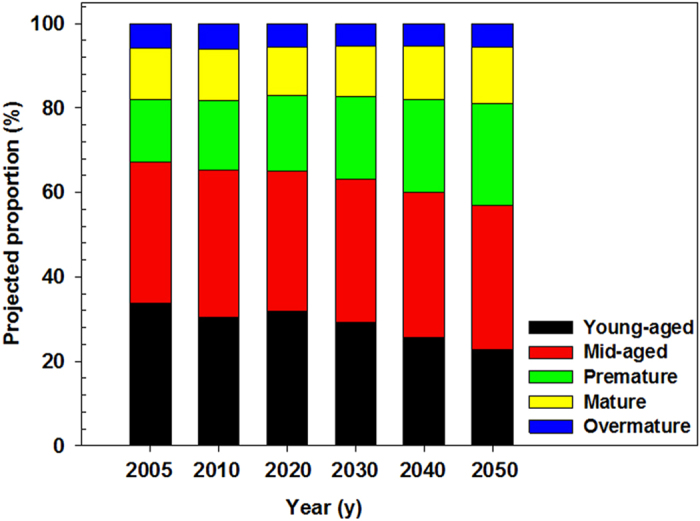
The projected proportions of stage-classified forest area based on our matrix model.

Despite the large newly planted forests (which were classified as young-aged stage), the proportion of young-aged forests decreased from 33.8% in 2005 to 22.8% in 2050 (Fig. 1). In contrast, the proportion of premature forests increased from 14.8% in 2005 to 24.3% in 2050. As a consequence, the area-weighted biomass C density of the total forests in China increased from 41.3 Mg C ha^−1^ in 2005 to 45.2 Mg C ha^−1^ (95% confidence interval: 40.7 ~ 50.2 Mg C ha^−1^) in 2050. Note that although China’s forests had low biomass C density (41.8 Mg C ha^−1^), they could have a relatively large biomass C sequestration capacity during 2010–2020 due to the large area increment (about 24 × 10^6^ ha) in this period.

The increase in both total area and average biomass C density suggests that China’s forests will function as a C sink in the next 45 years. Total forest biomass C stock will increase by 55.2% (39.7 ~ 72.3%) from 6.43 Pg C in 2005 to 9.97 Pg C (8.98 ~ 11.07 Pg C) in 2050, with a net accumulation of 3.55 Pg C (2.55 ~ 4.65 Pg C) (Table 2). Annual forest biomass C sink averaged 78.8 Tg C yr^-1^ (56.7 ~ 103.3 Tg C yr^−1^) between 2005 and 2050, with the minimum of 57.2 Tg C yr^−1^ (35.4 ~ 82.6 Tg C yr^−1^) in 2005-2010 and the maximum of 92.0 Tg C yr^−1^ (70.7 ~ 115.9 Tg C yr^−1^) in 2010 s.

### Backward projection for area and biomass C stock of China’s forests

**Figure 2:**
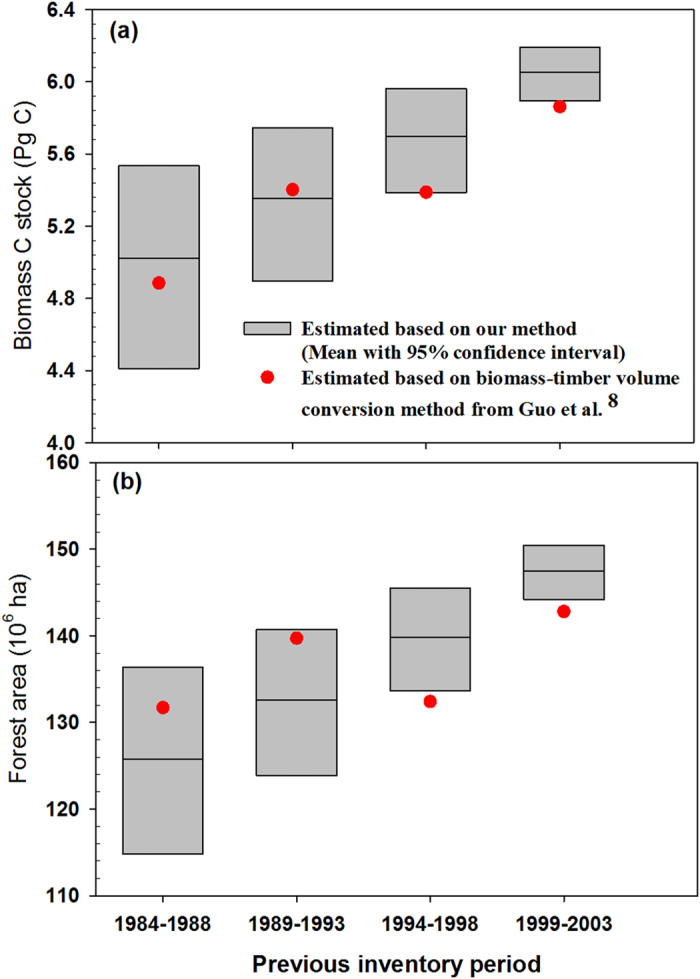
Comparing predicted biomass C stock (**a**) and forest area (**b**) during 1984-2003 based on the biomass-timber volume conversion method given by Guo *et al*. 8 and our estimations.

Based on the transition matrix, we performed a backward projection to estimate forest area and forest biomass C stock in China for the past four inventory periods (1984–1988, 1989–1993, 1994–1998, and 1999–2003) (Fig. 2; also see Supplementary Table S2 online). Our model predicted total forest area of 125.8 × 10^6^, 132.6 × 10^6^, 139.8 × 10^6^, and 147.5 × 10^6^ ha in 1984–1988, 1989–1993, 1994–1998, and 1999-2003, respectively. The area-weighted biomass C density was predicted to increase from 39.9 Mg C ha^−1^ in 1984–1988 to 41.0 Mg C ha^−1^ in 1999–2003 (see Supplementary Table S2 online). Finally, total forest biomass C stock increased consistently from 5.02 Pg C in 1984–1988 to 6.05 Pg C in 1999–2003 (Fig. 2a; also see Supplementary Table S2 online).

## Discussion

The past decades have seen growing interest in estimating the dynamics of forest biomass C stocks at the national and regional scales^5^,^6^,^7^,^8^,^16^. However, a general approach has yet to be developed to predict the future changes in forest biomass C stocks^11^,^17^,^18^. Here, we employed a stage-classified matrix approach to predict forest biomass C stocks of China’s forests in the next 45 years. To our knowledge, our study is the first attempt to apply stage-classified matrix models for the forest C dynamic studies. The derived transition matrix provides a country-level description for China’s forest dynamics, and facilitates projections for future C stocks. We predict a net increase of forest C storage by 3.55 Pg C during the period 2005–2050. Without considering the influences of forest dieback and harvesting, Xu *et al*.^11^ predicted a much larger C sink (7.23 Pg C) based on the relationships between biomass density and forest age for 36 forest types in China. However, Guo^19^ estimated that forest dieback could release C of 12.4 ~ 25.9 Tg C yr^−1^ during the period of 1984–2003. Because the transition matrix was derived from the most updated data of three inventory periods (1994–1998, 1999–2003 and 2004–2008), and involved the influence of historic natural and human disturbances, our results should provide an improved projection for China’s forest biomass C stocks.

The reliability of our matrix-based approach is evidenced by a comparison between our backward prediction for biomass C stocks during 1984–2003 and the estimates induced from the biomass-timber volume conversion method by Guo *et al*.^8^ (Fig. 2a). Our estimates about total forest area and biomass C stocks are generally consistent with those in Guo *et al*.^8^, indicating that our approach is reasonable at the national scale. However, there are indeed derivations in specific periods. Our predicted C stocks were greater (2.8%, 5.7% and 3.3%) in 1984–1988, 1994–1998 and 1999–2003, while smaller (−0.9%) in 1989–1993, than those corresponding estimates made by Guo *et al*.^8^. Forest area used in these two studies could explain most of the differences in biomass C stocks (Fig. 2b). Compared with those corresponding values reported by Guo *et al*.^8^, our estimates of forest area were smaller (−0.9%) in 1989-1993 and greater (5.6% and 3.3%) in 1994–1998 and 1999–2003, which followed the same trend as our estimates of biomass C stocks. In addition, as the newly planted forest area varied among different inventory periods, using a constant parameter for the newly planted forest area in the transition matrix could also induce derivations in the backward estimation of biomass C stocks.

Several factors could induce uncertainties in our projections. One of the major uncertainties may be caused by the estimation of the matrix parameters because we used the matrix parameters from the same transition matrix to project the dynamics of forest area in all provinces (see Methods section). Actually, forests in different provinces are dominated by different species, and thus have large variations in their life history and stage classification (see Supplementary Table S3 online). This suggests that forest dynamics should be hard to follow the same transition matrix across all the provinces. In our study, we used the bootstrap techniques to assess the uncertainty arising from this calculation. Additionally, there are other assumptions which also induce uncertainties, but are not easy to evaluate. First, to estimate the transition matrix parameters, we assumed that the newly planted forest area in a province was proportional to the total forest area of this province in the previous inventory period. However, in practice the newly planted forest area in each province should be affected by the availability of the suitable forest lands and the input of local people. Second, we assumed that parameters of the transition matrix would stay unchanged in the future, but many natural or human disturbances could affect these parameters, such as the unpredictable outbreaks of fire, insects, drought, and harvesting. Third, we used the stage-classified biomass C densities for the inventory period 2004-2008 to predict biomass C stocks in China’s forests up to 2050. In fact, our previous study showed that such stage-specific biomass C densities also varied among different inventory periods^8^. Finally, in addition to model-induced uncertainties, biomass C accumulation in forests can also be influenced by natural factors, such as climate change, elevation of atmospheric CO_2_, and N deposition^20^,^21^,^22^,^23^, which would be difficult to be incorporated into this study. Despite these uncertainties, our stage-classified modeling approach provides a promising tool for studying forest dynamics and predicting future changes in forest biomass C stocks. Future studies may incorporate more detailed information into the model to reduce these uncertainties.

## Methods

### Datasets

In this study, we used China’s forest inventory data and planned forest coverage data up to 2050. The forest inventory was conducted every five years since 1980 s for a total of five periods: 1984–1988, 1989–1993, 1994–1998, 1999–2003, and 2004–2008^24^,^25^,^26^,^27^,^28^. According to the inventory, forests were divided into three major categories: forest stands (including planted and natural forests), economic forests, and bamboos. As forest stands are the major component of China’s forests and documented in some details by stage class in each inventory period, we only considered forest stands in our study. Besides, due to the lack of data, forest stands in Hong Kong, Macao, and Taiwan were not included in our study.

Forest stands were divided into five stage classes based on their growth stage: young-aged, mid-aged, premature, mature and overmature forests (see Supplementary Table S3 online). The inventory documented detailed information on the areas and timber volumes by dominant tree species and by stage class in each province.

Based on the development goals set by the China Forestry Sustainable Development Strategy Research Group^29^, we derived the expected total forest area in the 45 years of 2005–2050 (Table 3). To obtain the area of forest stands, we assumed that the fraction of forest stands to total forests in the next 45 years will be the same as during the period of 1999–2008 (81.0%). We therefore obtained net increase of forest stand area from the difference in total forest stand area between any two sequential periods (Table 3).

### Stage-classified matrix models

**Figure 3:**
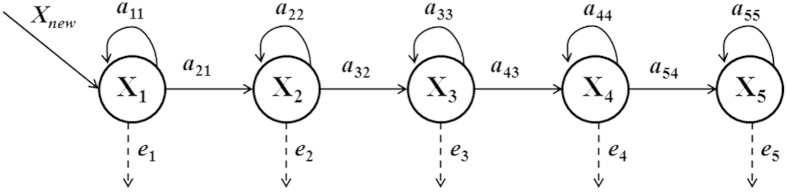
The dynamics of forest area in our stage-classified matrix model. *X*_*new*_*, X*_*1*_, *X*_*2*_, *X*_*3*_, *X*_*4*_, and *X*_*5*_ represent the forest area of newly planted, young-aged, mid-aged, premature, mature, and overmature forests in inventory, respectively. a_*21*_, a_*32*_, a_*43*_, and a_*54*_ represent the transition probability from the young-aged class to the mid-aged class, the mid-aged class to the premature class, the premature class to the mature class, and the mature class to the overmature class between two sequential inventory periods, respectively. a_*11*_, a_*22*_, a_*33*_, a_*44*_, and a_*55*_ represent the probability for the young-aged, mid-aged, premature, mature, and overmature classes to remain within itself between two sequential inventory periods, respectively. *e*_*1*_, *e*_*2*_, *e*_*3*_, *e*_*4*_, and *e*_*5*_ represent the probability of the young-aged, mid-aged, premature, mature, and overmature forests in the corresponding stage to die or be harvested, respectively.

We used a five-stage transition matrix to model the dynamics of China’s forests. We assumed that between two sequential inventories, for forests at one stage class, one part would remain in its stage class, another part would transfer to the next stage class, and the rest would die or be harvested (Fig. 3). We only considered transitions between neighbor stages because China’s forest inventory is conducted every five years, and the age span of all stage classes for all dominant species are generally longer than or equal to 5 years (see Supplementary Table S3 online). Finally, differing from population dynamics where population growth is caused by reproduction, in our case the growth of forest area is always achieved by plantation. Therefore, the dynamics of forest area are modeled as follows:


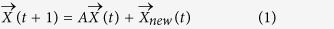


where


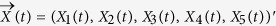



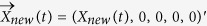



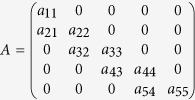


Subscripts *t* and *t+1* represent two sequential inventory periods. *X*_*new*_*(t), X*_*1*_*(t)*, *X*_*2*_*(t)*, *X*_*3*_*(t)*, *X*_*4*_*(t)*, and *X*_*5*_*(t)* represent the forest area of newly planted, young-aged, mid-aged, premature, mature, and overmature forests in inventory period *t*, respectively. In matrix *A*, *a*_*ij*_ represents the transition probability from the *j-th* stage class to the *i-th* stage class between two sequential inventory periods. In particular, *a*_*jj*_ represents the probability for the *j-th* stage class to remain within itself between two sequential inventory periods. Note that all parameters in the matrix *A* should lie between 0 and 1, and the sum of any column of *A* should be no larger than 1, where the difference (*e*_i_ = 1−*a*_*ii*_*−a*_*i,i+1*_) indicates the probability of forests in the corresponding stage to die or be harvested (Fig. 3).

### Parameter estimation and projection

Three assumptions were made to estimate the parameters in matrix *A*: (1) in all provinces, forest dynamics follow the same transition matrix; (2) for any stage class, the probabilities of maintaining, transferring, and dying between any two sequential inventory periods do not vary through time (in other words, the transition matrix stays unchanged during our study period); and (3) the area of newly planted forests in a province is proportional to its current total forest area (e.g., the area of newly planted forests from 1999–2003 to 2004–2008 in Beijing is proportional to the total forest area in 1999–2003 in Beijing). With these assumptions, estimating the transition matrix relies on finding parameters that minimize the square derivations between the inventoried forest area and those predicted from the previous inventory (see Supplementary Text S1online).

Because the definition of forest stands in China’s forest inventory has been changed from >30% to ≥20% canopy coverage since 1994, we only used forest inventory data from the periods of 1994–1998, 1999–2003, and 2004–2008 for the parameter estimation. Each pair of two sequential inventories of each province provides a sample. As a result, we initially had 60 samples (30 provinces × 2 inventory pairs). Due to the lack of detailed stage-classified forest area data for the Tibetan Plateau during 1994–1998, we finally used 59 samples.

The estimated transition matrix was used to project the changes of China’s forest area in each stage class from 2005 to 2050. Starting with the stage-classified forest area during the inventory period 2004–2008 (serving as the start point of 2005), we estimated forest area of each stage class every five years up to 2050. Based on the inventory data in 2004–2008, we also performed a backward estimation for forest area during the past four inventory periods (1984–1988, 1989–1993, 1994–1998 and 1999–2003). For doing it, we retained the assumption that the newly planted forest area is proportional to the standing forest area during these periods and the proportion is the same as that in 1994–2008. Finally, to estimate biomass C stock in China’s forests based on the projected area, we used the stage-classified biomass C densities for the inventory period 2004–2008 (the biomass densities for young-aged, mid-aged, premature, mature and overmature forests in 2004–2008 are 18.9, 37.1, 54.6, 72.8 and 96.4 Mg C/ha, respectively).

A bootstrap approach was employed to evaluate the uncertainty in matrix parameters and projections of forest area and biomass C stocks. Specifically, we first sampled (with replacement) equal number of samples from the original 59 data, and then estimated the transition matrix parameters and predicted the changes of forest area and biomass C stock. The procedures were repeated 10,000 times. The 2.5% and 97.5% quantiles of the 10000 values give the lower and upper boundaries (95% confident interval) for the corresponding properties.

## Additional Information

**How to cite this article**: Hu, H. *et al*. The stage-classified matrix models project a significant increase in biomass carbon stocks in China's forests between 2005 and 2050. *Sci. Rep*. **5**, 11203; doi: 10.1038/srep11203 (2015).

## Supplementary Material

Supplementary Information

## Figures and Tables

**Table 1:** Parameter estimates of the transition matrix.

Age class	Young-aged	Mid-aged	Premature	Mature	Overmature
Young-aged	0.8557	0	0	0	0
Mid-aged	0.1442	0.9205	0	0	0
Premature	0	0.0792	0.9541	0	0
Mature	0	0	0.0447	0.9821	0
Overmature	0	0	0	0.0144	0.9992
Dead or harvested	0.0002	0.0003	0.0013	0.0036	0.0008

The transition probabilities are calculated as the average of 10000 bootstrap re-sampling. The dead or be harvested probabilities are derived as follows: *Pr* (dead or be harvested for stage *i*) = 1- *Pr* (remaining within itself for stage *i*)- *Pr* (transferring into the next stage for stage *i*). For details, see Supplementary Table S1 for 95% confident interval for each parameter.

**Table 2:** Biomass C stock, C density and C sink of China’s forests between 2005 and 2050.

Year	C stock (Pg C)	C density (Mg C ha^−1^)	C sink (Tg C yr^−1^)
2005*	6.43	41.3	
2010	6.71 (6.60 ~ 6.84)	42.3 (41.7 ~ 43.1)	57.2 (35.4 ~ 82.6)
2020	7.63 (7.31 ~ 8.00)	41.8 (40.0 ~ 43.8)	92.0 (70.7 ~ 115.9)
2030	8.46 (7.91 ~ 9.08)	42.6 (39.8 ~ 45.7)	83.1 (60.1 ~ 108.0)
2040	9.22 (8.44 ~ 10.08)	44.0 (40.3 ~ 48.1)	75.5 (52.8 ~ 100.1)
2050	9.97 (8.98 ~ 11.07)	45.2 (40.7 ~ 50.2)	75.5 (53.8 ~ 99.5)
2005–2050			78.8 (56.7 ~ 103.3)

Means are followed by 95% confidence interval in parenthesis for the predicted values.

^*^Data from Guo *et al*.^8^ for the inventory period of 2004-2008.

**Table 3:** Forest area by 2050 in China.

Year	Coverage (%)	Area (10^4^ ha)		
		Total forests	Forest stands	Net increase in the area of forest stands
2005*	20.1	19333	15559	
2010	20.4	19568	15856	297
2020	23.5	22529	18255	2399
2030	25.5	24503	19855	1600
2040	26.9	25865	20958	1103
2050	28.4	27227	22061	1103

Total forest area was derived from the forest coverage plan and total land area of China (960.27 × 10^6^ ha). We subtracted the area of special shrubs and forests in Hong Kong, Macao, and Taiwan from the total forest area.

Coverage data are based on China Forestry Sustainable Development Strategy Research Group^28^

^*^Data from the inventory period of 2004–2008.
