# Supplementary material for: The stage-classified matrix models project a significant increase in biomass carbon stocks in China’s forests between 2005 and 2050
Source: Sci Rep. 2015 Jun 25;5:11203. doi: 10.1038/srep11203 (PMC4480144; doi:10.1038/srep11203)
Supplement: Supplementary Information [file srep11203-s1.doc]

**Online Supporting Information**

**The stage-classified matrix models project a significant increase in biomass carbon stocks in China’s forests between 2005 and 2050**

Huifeng Hu,^1†^ Shaopeng Wang,^2,4†^ Zhaodi Guo,^2,3^ Bing Xu^2^ & Jingyun Fang[^1,2*^](mailto:fangjingyun@ibcas.ac.cn)1,2*)

^1^ State Key Laboratory of Vegetation and Environmental Change, Institute of Botany, Chinese Academy of Sciences, Beijing, China, ^2^Department of Ecology, College of Urban and Environmental Science, and Key Laboratory for Earth Surface Processes of the Ministry of Education, Peking University, Beijing, China, ^3^National Satellite Meteorological Center, China Meteorological Administration, Beijing, China.

^4^Current address: Centre for Biodiversity Theory and Modelling, Station d’Ecologie Experimentale du CNRS, Moulis, France.

† These authors contributed equally to this work.

**^*^ Corresponding author:**

Jingyun Fang

Professor

Department of Ecology, Peking University

Beijing 100871, China

Tel/Fax: +86 10 6275 6560;

E-mail: [fangjingyun@ibcas.ac.cn](mailto:fangjingyun@ibcas.ac.cn); or [jyfang@urban.pku.edu.cn](mailto:jyfang@urban.pku.edu.cn) (J.Y. Fang).

This file contains Table S1-S3, Text S1, and references.

Table S1 | 95% confident interval for each parameter in the transition matrix, obtained by the 2.5% and 97.5% quantiles of 10000 bootstrap re-sampling

|  | Young-aged | Mid-aged | Premature | Mature | Overmature |
| --- | --- | --- | --- | --- | --- |
| Young-aged | (0.776 ~ 0.924) | 0 | 0 | 0 | 0 |
| Mid-aged | (0.077 ~ 0.224) | (0.878 ~ 0.958) | 0 | 0 | 0 |
| Premature | 0 | (0.042 ~ 0.119) | (0.876 ~ 1) | 0 | 0 |
| Mature | 0 | 0 | (0 ~ 0.116) | (0.930 ~ 1) | 0 |
| Overmature | 0 | 0 | 0 | (0 ~ 0.038) | (0.992 ~ 1) |

Table S2 | Backward projection of forest area and biomass C stock of China’s forests in the past two decades. Means are followed by 95% confidence interval in parenthesis for the predicted values

| Period | Forest area (10^6^ ha) | C stock (Pg C) | C density (Mg C ha^-1^) |
| --- | --- | --- | --- |
| 1984-1988 | 125.8 (114.8~136.3) | 5.02 (4.41~5.53) | 39.9 (38.4~40.6) |
| 1989-1993 | 132.6 (123.9~140.7) | 5.35 (4.89~5.74) | 40.4 (39.5~40.8) |
| 1994-1998 | 139.8 (133.6~145.5) | 5.70 (5.39~5.96) | 40.7 (40.3~41.0) |
| 1999-2003 | 147.5 (144.2~150.5) | 6.05 (5.90~6.19) | 41.0 (40.9~41.1) |

Table S3 | Classification of forest ages for different dominant tree species ^*^

| Dominant tree species | Region | Origin | Age class (years) | | | | | | | Age span  (years) |
| --- | --- | --- | --- | --- | --- | --- | --- | --- | --- | --- |
|  |  |  | Young-aged | Mid-aged | | Premature | | Mature | Overmature |  |
| *Pinus koraiensis, Picea, Cupressus, Tsuga* | North | Natural | <60 | 61–100 | 101–120 | | 121–160 | | 161 | 20 |
|  | North | Planted | <40 | 41–60 | 61–80 | | 81–120 | | 121 | 20 |
|  | South | Natural | <40 | 41–60 | 61–80 | | 81–120 | | 121 | 20 |
|  | South | Planted | <20 | 21–40 | 41–60 | | 61–80 | | 81 | 20 |
| *Larix, Abies, P. sylvestris* var*. mongolica, P. densiflora, P. thunbergii* | North | Natural | <40 | 41–80 | 81–100 | | 101–140 | | 141 | 20 |
|  | North | Planted | <20 | 21–30 | 31–40 | | 41–60 | | 61 | 10 |
|  | South | Natural | <40 | 41–60 | 61–80 | | 81–120 | | 121 | 20 |
|  | South | Planted | <20 | 21–30 | 31–40 | | 41–60 | | 61 | 10 |
| *P. tabulaeformis, P. massoniana, P. yunnanensis, P. kesiya* var. *langbianensis, P. armandii, P. densata* | North | Natural | <30 | 31–50 | 51`60 | | 61–80 | | 81 | 10 |
|  | North | Planted | <20 | 21–30 | 31–40 | | 41–60 | | 61 | 10 |
|  | South | Natural | <20 | 21–30 | 31–40 | | 41–60 | | 61 | 10 |
|  | South | Planted | <10 | 11–20 | 21–30 | | 31–50 | | 51 | 10 |
| *Populus, Eucalyptus, Sassafras, Paulownia, Casuarina*, etc. | North | Planted | <10 | 11–15 | 16–20 | | 21–30 | | 31 | 5 |
|  | South | Planted | <5 | 6–10 | 11–15 | | 16–25 | | 26 | 5 |
| *Betula, Ulmus, Schima, Liquidambar, Davidia* | North | Natural | <30 | 31–50 | 51–60 | | 61–80 | | 81 | 10 |
|  | North | Planted | <20 | 21–30 | 31–40 | | 41–60 | | 61 | 10 |
|  | South | Natural | <20 | 21–40 | 41–50 | | 51–70 | | 71 | 10 |
|  | South | Planted | <10 | 11–20 | 21–30 | | 31–50 | | 51 | 10 |
| Oaks, *Cinnamomum, Phoebe, Tilia, Fraxinus mandschurica, Juglans mandshurica, Phellodendron,* etc. | North & South | Natural | <40 | 41–60 | 61–80 | | 81–120 | | 121 | 20 |
|  | North & South | Planted | <20 | 21–40 | 41–50 | | 51–70 | | >71 | 10 |
| *Cunninghamia, Cryptomeria, Metasequoia* | South | Planted | <10 | 11–20 | 21–25 | | 26–35 | | >36 | 5 |

^*^ Xiao, X.W. *Forest Resource Inventory of China* (China Forestry Publishing House, Beijing, 2005).

**Text S1. Parameter estimation of the transition matrix**

For each province, forest dynamics between every two adjacent inventory periods can be described by the following transition matrix (see main text for definition):

$\overrightarrow{X}(t+1)=A\overrightarrow{X}(t)+\overrightarrow{X}_{new}(t)$ (1)

We then define an error function as follows:

$R(a_{11},a_{21},a_{22},a_{23},a_{33},a_{34},a_{44},a_{45},a_{55},\lambda)=\sum_{i=1}^{59} \left\|\overrightarrow{X}_{i}(t+1)-[A\overrightarrow{X}_{i}(t)+\overrightarrow{X}_{newi}(t)]\right\|^{2}$ (2)

Here, ||..|| denotes the Euclidean norm of a vector (square root of the sum of the squares of all elements in the vector). *i* corresponds to the *i*-th sample (area vector pair for a province). Specifically, *i* = 1, ..., 29 denotes the area vector pair between periods 1994-1998 and 1999-2003 for 29 provinces (except Tibet, because there is no detailed information on the area for each stage class of the whole Tibet’s forests in 1994-1998), and *i* = 30, ..., 59 denotes the area vector pair between periods 1999-2003 and 2004-2008 for 30 provinces. Therefore, the error function is calculated as the sum of squared errors between observed and (transfer matrix-based) predicted area for each stage and for each province.

To facilitate estimation of the transition matrix, the newly planted forest area for each province is assumed to be a fixed proportion (λ) of its original total area. For instance, from 1999-2003 to 2004-2008, the newly planted forest area in any province is a proportion (λ) of the total forest area in this province in 1999-2003. With this assumption, parameter estimation is to find the parameters that minimize the error function. It is noted that all parameters of the transfer matrix *A* should be within [0, 1] and the column sums of *A* are all less than or equal to 1. We solved this constrained optimization problem by using the function "solve.QP" in package "quadprog" in software *R*.
